# Guts Imbalance Imbalances the Brain: A Review of Gut Microbiota Association With Neurological and Psychiatric Disorders

**DOI:** 10.3389/fmed.2022.813204

**Published:** 2022-03-31

**Authors:** Laura Mitrea, Silvia-Amalia Nemeş, Katalin Szabo, Bernadette-Emőke Teleky, Dan-Cristian Vodnar

**Affiliations:** ^1^Institute of Life Sciences, University of Agricultural Sciences and Veterinary Medicine of Cluj–Napoca, Cluj-Napoca, Romania; ^2^Faculty of Food Science and Technology, University of Agricultural Sciences and Veterinary Medicine of Cluj–Napoca, Cluj-Napoca, Romania

**Keywords:** gut microbiota, microbiome, gut-brain axis, dysbiosis, neuropsychiatric affections, mental health, psychobiotics

## Abstract

Over the last 10 years, there has been a growing interest in the relationship between gut microbiota, the brain, and neurologic-associated affections. As multiple preclinical and clinical research studies highlight gut microbiota’s potential to modulate the general state of health state, it goes without saying that gut microbiota plays a significant role in neurogenesis, mental and cognitive development, emotions, and behaviors, and in the progression of neuropsychiatric illnesses. Gut microbiota produces important biologic products that, through the gut-brain axis, are directly connected with the appearance and evolution of neurological and psychiatric disorders such as depression, anxiety, bipolar disorder, autism, schizophrenia, Parkinson’s disease, Alzheimer’s disease, dementia, multiple sclerosis, and epilepsy. This study reviews recent research on the link between gut microbiota and the brain, and microbiome’s role in shaping the development of the most common neurological and psychiatric illnesses. Moreover, special attention is paid to the use of probiotic formulations as a potential non-invasive therapeutic opportunity for prevention and management of neuropsychiatric-associated affections.

## Graphical Abstract

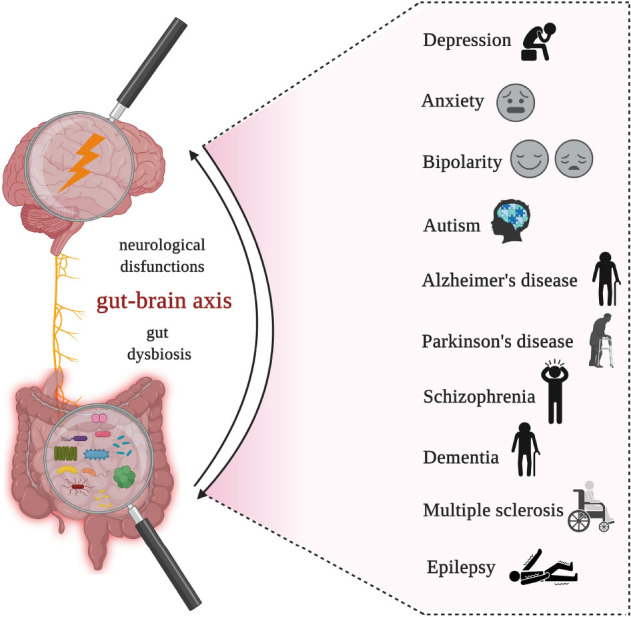

## Brief Introduction

Gut microbiota are a very intricate ecosystem that shelter an abundant and multifarious community of microbes that evolve in a human host in a reciprocal relationship. At the same time, gut microbiome sums up all genomic characteristics of gut microbes that are closely linked with the health status of the host. Moreover, as the human gastrointestinal tract is a generous environment comprising over 100 trillion microbes, this aspect makes the microbiome the great “virtual organ” of the body, which consequently influences and modulates the host’s fitness, phenotype, and health (1). Dysbiosis, which is known as disequilibrium in this complex ecosystem, is responsible for a variety of human illnesses that may manifest at every physiological system level (2). Even though the notion of “dysbiosis” is a broad term used lately as a mental shortcut, intestinal dysbiosis is more and more associated with unwholesome microbiota and pathogenesis of both gut-related and extra-intestinal affections (3). At the same time, the complex nature of the gut microbiota is also particular to every individual organism and is extremely responsible for both well state of an individual, and unhealthy outcomes when pathogenic microbes known as pathobionts are expended (4). Moreover, metabolic and nutritional homeostasis, immune system functioning, intestinal barrier integrity, and cerebral activity are all influenced and modulated by the gut microbiota, which directly impacts the crucial physiological functions of the host (5). It seems that any discrepancy in gut microbiota and host communication can be considered as a triggering element in the pathogenesis of diseases (6–9).

Given that the incidence of neuropsychiatric-associated affections is increasing and the impact of gut microbiota in their development is highly studied (10), in this review article, we discuss the role of microbiota in the most frequent mental-associated illnesses including depression, anxiety, bipolar disorders, autism, schizophrenia, Parkinson’s disease, Alzheimer’s disease, dementia, multiple sclerosis, and epilepsy as a consequence of gut dysbiosis. Taking notice of recent review articles that are focused on the impact of gut microbiota on one or few specific neurological illnesses, this article covers a large spectrum of neuropsychiatric diseases like those mentioned above. In this context, we analyze scientific literature published over the last decade that examines the role of gut microbiota in the development of the most common mental-associated disorders, focusing on the consequences of both gut balance/disequilibrium and diseases. Moreover, as microbes are key signaling components in bidirectional communication of the gut-brain axis, in this review, we investigated the intercommunication between the central nervous system (CNS) and the digestive system, and its contribution to mental health status. Last but not least, the use of probiotics as a potential non-invasive therapeutic opportunity for neuropsychiatric affections was also debated.

## Gut Microbiota and Neuropsychiatric Status Are Modulated by Extrinsic and Intrinsic Factors

Health and neuropsychiatric status are shaped by some extrinsic and intrinsic factors, such as lifestyle habits, dietary factors, and medicament intake. In terms of lifestyle habits, these exert a strong influence on gut microbiota and brain relationship and are included in the multifactorial pathogenesis of psychiatric disorders along with genetics, inflammation, and neurotransmitter imbalance. It is generally accepted that physically active people have less risk of developing cognitive impairment and higher cognitive performance. Other important lifestyle-related habits with a great influence on the development of gut dysbiosis and, consequently, onset of brain function disorders are oral hygiene, nicotine abuse, and sleep deprivation (11–14).

Dietary factors are also crucial in health wellbeing. Intrinsic human feeding routine is influenced by culture, religion, and society. It shapes cognitive capacity and brain evolution, and it empowers dietary habits to modulate mental health at both the individual and population-wide levels. Therefore, nutrition can sustain homeostasis or contribute significantly to disease development. A high fat diet (HFD), composed predominantly of saturated and/or trans fats, seems to modify the microbiota by over-representation of lipopolysaccharide (LPS)-expressing bacteria, leading to elevated levels of LPS in the systemic circulation and pro-inflammatory state of the host, and reduced synaptic plasticity (15–17). Balanced intestinal microbiota stimulate a regulatory milieu in the gut-associated lymphoid tissue (GALT) through the production and release of various immunomodulatory compounds, like short-chain fatty acids (SCFAs), and help in the prevention of the development of various immune-mediated and neurological disorders (18, 19). SCFA production can be shaped by intake of prebiotics and probiotics, or by adherence to the Mediterranean diet that consists mainly of whole grains, legumes, nuts, and fresh vegetables and fruits. Moderate consumption of meat, poultry, and fish provides fermentable substrates for probiotic bacteria. Various findings show that adherence to the Mediterranean diet reduces the incidence of clinical depression especially because it is high in vitamin B, which is further associated with the synthesis of important neurotransmitters such as serotonin, dopamine, and noradrenaline (20). Moreover, the mechanism through which the Mediterranean diet is linked with the modulation of mood and behavior is related to the advancement of monoamine neurotransmitter turnover (20).

Neuropsychiatric status is also influenced by various factors of biological or chemical origin. In biological terms, mental health can be disturbed by some physiological malfunctions/illnesses, such as obesity, diabetes mellitus, impaired lung function, and urological and genital diseases (21, 22). In chemical terms, particular exogenous compounds like medicaments (e.g., drugs, antibiotics) may significantly influence mental disorders’ appearance and development (6). For example, a recent meta-analysis on the impact of commonly used medications on modulation of the gut microbiota brings evidence for extensive changes in taxonomy and metabolic potential, and associations with functional changes in the gut. In this context, 19 of 41 analyzed drugs were found to be related to microbial features, and proton-pump inhibitors, metformin, antibiotics, and laxatives showed the strongest associations in the gut microbiome and brain functioning, as abuse or misuse of these chemical substances provides extensive changes in resistome profiles (23).

In the context of drug intake, antibiotics are probably the most used medications for multiple affections. As antibiotics are invaluable weapons for fighting infectious diseases, these chemicals alter both the gut microbiota and microbiome, leading to immune dysregulation. Intake of antibiotics profoundly affects the composition and function of the gut microbiota by disrupting the equilibrium among commensal populations, leading to long-lasting adverse effects on the host (24, 25). Administration of antibiotics in high dosage or for long periods can induce severe alterations or irreversible damages at both intestinal and brain levels (26, 27), while administration of antibiotics on a short-term basis is associated with weakened cognitive performance (28, 29). A particular study on adult mice (29) proved that antibiotic-based treatment determines alterations in the microbial community, and alters metabolites profiles from the colon and plasma. Also, the same study shows that antibiotics (e.g., ampicillin, bacitracin, meropenem, neomycin, and vancomycin) harm object recognition, and that the expression of neuronal signaling molecules such as brain-derived neurotrophic factor, N-methyl-d-aspartate receptor subunit 2B, serotonin transporter, and neuropeptide Y system in distinct brain areas was strongly disturbed in the adult mice (29). Antibiotic treatments unbalance gut microbial diversity; for instance, Firmicutes and Actinobacteria classes are quickly replaced by Proteobacteria and Bacteroidetes, an aspect correlated with a decreased level of SCFAs in the colon, which is further associated with neurological and brain dysfunctions (18, 25, 30–32). Some broad-spectrum antibiotics (e.g., metronidazole, penicillins, macrolides, sulfonamides, cephalosporins, and quinolones) that are administered for both gastrointestinal and other types of infections cross the blood-brain barrier and penetrate, to some extent, in brain tissues favoring neurological deterioration (30, 33). In a review article, Bhattacharyya et al. underscored that some antibiotic neurotoxic effects are seizures, psychosis (delusions, hallucinations), and mania (34). Additionally, gut dysbiosis induced by antibiotic therapies in early life may have an unfavorable neurological impact on adult age, and it also has a favorable impact on the development of illnesses like irritable bowel syndrome (IBS), allergies, obesity, and diabetes (18, 35–39). Furthermore, long-term exposure to antibiotic treatments is connected to a significant alteration in levels of neuromodulators that interact along the gut-brain axis, which is translated later on to cognitive deficits, altered dynamics of the tryptophan metabolic pathway, and significantly reduced brain-derived neurotropic factor, oxytocin, and expression of vasopressin in the brain of adults (35, 40). That is to say, the available clinical data point out that antibiotic treatments induce gut microbiota disturbances in various pathways, from temporary short-term changes to permanent long-term changes (27). Moreover, in terms of mental health, clinical studies reveal that gut microbiota alterations triggered by antibiotic use are closely connected with decreased hippocampal neurogenesis, memory retention, and object recognition impairment (7, 41).

## Bidirectional Connection Between the Gut and the Brain

Many studies have addressed the bidirectional communication between gut and brain, and the impact it has over the entire health state, including disorders that can develop from these relationship. This bidirectional connection is closely related to the sympathetic and parasympathetic branches of the autonomic nervous system (ANS), enteric nervous system (ENS), endocrine system (ES), and immune system (IS) (6, 31, 42–44).

The gut microbiome has a key role in influencing the development and function of the nervous system through its interaction with the gut-brain axis, and it has been suggested that a “microbiome-gut-brain axis” seems to be a more appropriate model, as it is responsible for a complex network of communication among the gut, microbial community from the intestine, and brain by modulating at the same time the gastrointestinal system, CNS, and IS (45, 46). The biochemistry behind the interconnection between the ENS and the CNS represented by the gut and the brain embraces many possible physiological pathways (47, 48). For instance, the neuronal circuits that transport signaling molecules, IS activation and its response to a possible pathogenic threat, production and release of targeted gut hormones by the ES, amino acid metabolism, and short-chain fatty acid (SCFA) biosynthesis. These mechanisms are interconnected and major factors responsible for the homeostasis of mental health (6, 42, 46). Nonetheless, the influence of gut microbiota in memory dysfunction has also been reported in studies performed on germ-free animals (49, 50). It was observed that the presence of microbiota modulated the serotoninergic system, with increases in serotonin turnover and modified levels of specific metabolites observed in the germ-free animals’ limbic system (50).

The gut microbiota can be profiled into three important bacterial enterotypes that are dominated by *Bacteroides*, *Prevotella*, and *Ruminococcus*, and the percentage for each of them is dietary and lifestyle-dependent (5, 51). Anyhow, the enterotype can be translated as the “stratification” of the gut microbiota, which gives at the same time particularity of the microbial community for each individual (52). Besides the role played in food and drug digestion, intestinal microbiota have an essential physiological role in motility, IS development (mucosal and systemic), water and fat absorption and distribution, xenobiotic metabolism, and biosynthesis of vitamin K and SCFAs (19, 53–57). SCFAs seem to have an important role in mediating gut-brain interactions owing to their neuroactive properties and their effects on other gut-brain signaling pathways including the IS and ES (58). SCFAs represented predominantly by acetate, propionate, butyrate, lactate, and succinate in the human body, are saturated fatty acids produced by fermentation of fiber-rich substrates by bacteria (e.g., *Ruminococcus*, *Bacteroides*, *Roseburia*, *Prevotella*), which influence immune cells and immune modulators to maintain homeostasis (19, 59). Last but not least, though the gut-brain links the human microbiome is directly involved in both the health and malfunction of the CNS, as it is a key regulator in neurogenesis, neurodevelopment, and behavior (5, 42, 51, 60–63). In addition, studies conducted on germ-free animals strengthen the idea of the fact that gut bacterial community is essential in the development of both ENS and CNS, and that the absence of microbes’ colonization is tightly linked with altered expression and turnover of neurotransmitters at both levels (50). At the same time, gut microbiota’s disturbances are correlated with alterations in gut sensory-motor functions translated through slowed-down gastric emptying and intestinal transit, decreased migrating motor complex cyclic recurrence and distal propagation, and enlarged cecal size (50).

It is generally understood that the major part of an individual’s microbial colonization occurs after birth, mainly originating from the maternal microbiota (64). There is a delicate balance between human microbiota and the development of different pathogenesis, modulated by external factors like diet, drug intake, and lifestyle habits (smoking, alcohol intake, and disrupted sleeping habits), which can easily influence the bacterial communities in the gut. Furthermore, bacterial metabolites from the gut, such as SCFAs, gamma-aminobutyric acid (GABA), and tryptophan, have a substantial influence on the regulation of the gut-brain axis. For example, even a short-term (5 days) consumption of diets composed entirely of animal or plant products alters microbial community structure affecting microbial gene expression among individuals (65, 66). Bacterial cluster responses to the animal-based diet showed an increased abundance of bile-tolerant microorganisms (*Alistipes*, *Bilophila*, and *Bacteroides*) and decreased levels of *Firmicutes* that metabolize dietary plant polysaccharides (*Roseburia*, *Eubacteriumrectale*, and *Ruminococcusbromii*). Fermentable dietary fiber, prebiotics, and probiotic-based diets contribute to the proliferation of SCFA-producing bacteria, which might influence gut-brain communication and brain function directly or indirectly through immune, endocrine, vagal, and other humoral pathways (58).

**FIGURE 1:**
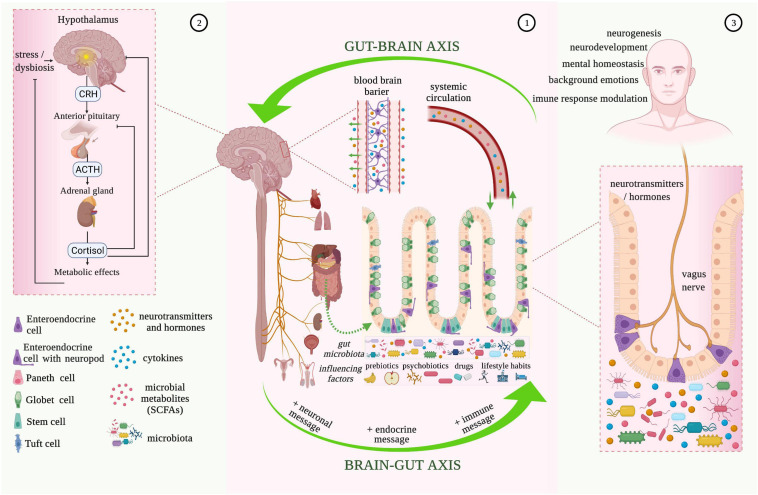
Microbiota-gut-brain bidirectional relationship. 1. The vagus nerve transfers information on the state of the digestive system to the brain through sensory fibers. It sends neuronal, endocrine, and immune messages to the gut microbiota influencing the state of brain health through various pathways (e.g., by bidirectional vagus nerve-to-brain communication, or through the enteric nervous system (ENS) and enteroendocrine cells with neuropods). Gastrointestinal bioactive molecules (neurotransmitters and hormones, cytokines, and microbial metabolites) also produced under the influence of external factors, such as prebiotics, psychobiotics, drugs, and lifestyle habits, end up in the brain tissue through the blood-brain barrier that consists of endothelial cells of the brain capillary wall, astrocyte end-feet surrounding the capillary, and pericytes embedded in the capillary basement membrane. 2. Hypothalamic-pituitary-adrenal (HPA) axis activation is characterized by the release of corticotropin-releasing hormone (CRH) from the hypothalamus, which then stimulates the delivery of adrenocorticotropic hormone (ACTH) from the anterior pituitary gland. ACTH acts on the adrenal gland to produce and release the stress hormone (cortisol), which is responsible for the modulation of the intestinal epithelial barrier and immune responses. 3. Enteroendocrine cells with neuropods are one of the most important influencers of bidirectional brain-gut communication. The innervation induced to the enterochromaffin-cell signaling to neuronal circuits by the vagus nerve, modulates the pain response, background emotions, immune-response, neurogenesis, and neurodevelopment. The vagus nerve also has immunomodulatory properties and plays an essential role in gastrointestinal and psychiatric disorders (e.g., depression, posttraumatic stress disorder, anxiety) (image created using the BioRender application; https://app.biorender.com).

The brain-gut axis is well-defined by the communication that exists between the cerebral cortex and the digestive system (50) (Figure 1). The brain-gut axis, in particular, consists of the brain, spinal cord, ANS, ENS, and hypothalamic-pituitary-adrenal (HPA) axis (67). The brain-gut axis’s most specific purpose is to allow signals engendered from the brain to influence the response given by intestinal microbiota and the visceral reaction transmitted to the brain (68). Brain-gut axis disturbances are the main cause of the most frequent gastrointestinal symptoms and syndromes, such as IBS, functional dyspepsia, functional biliary pain, chronic abdominal pain, gastroparesis, constipation, gastroesophageal reflux disease (GRD), and cyclic vomiting syndrome (CVS) (67, 69). Gastrointestinal motility disorders act through abnormalities of the neurenteric system, which causes delayed or fast transit.

The connection between the brain and bowel is made through neural pathways alongside immune and endocrine mechanisms (70). Information from periphery receptors in the intestinal tract is transferred to the CNS by neural pathways, to the hypothalamus especially, where it is assimilated and evaluated at cortical levels, and the response generated is sent back, following actions of the ENS (43). The hypothalamus is part of the limbic system, besides the amygdala, medial thalamus, and anterior cingulate cortex, which is responsible for “brain-body interaction.” The limbic system’s primary functions are represented by monitoring and responding to internal and external factors (pain, fear, anger, curiosity, lethargy) and by processing social signals of emotion (posture and facial expression) (60). The ANS maintains control at the bowel level with sympathetic and parasympathetic subdivisions. In terms of the sympathetic nervous system, this innervates the intestinal area through prevertebral ganglia by efferent routes of splanchnic nerves. Furthermore, sympathetic nervous system innervation is responsible for the constriction of intestinal and urinary sphincters, and it is also correlated with a reduction in intestinal activity (67). The vagus nerve (tenth cranial nerve) is part of the cranial nervous system, more exactly the parasympathetic nervous system, alongside nervus oculomotorius, nervus facialis, and nervus glossopharyngeus. It has high relevance in the brain-gut communication for being responsible for the control of emotional state, immune response, digestion, heart rate, and respiratory rate (67, 71). The vagus nerve represents the principal pathway from the gut cavity to the brain and innervates the pharynx and larynx, where it is responsible for swallowing and vocalization, and visceral organs (67). Sensory fibers from the vagus nerve transfer information on the state of inner organs (heart, lung, pancreas, stomach, liver, and intestines) to the brain (80–90% of fibers) and vice versa, from the brain to organs (10–20% of fibers) (71). The vagus nerve also acts synergistically with several neurotransmitters (e.g., oxyntomodulin, cholecystokinin, ghrelin) released from the ENS and controls food intake and appetite (71, 72). The vagus nerve also has immunomodulatory properties and plays an essential role in gastrointestinal and psychiatric disorders (e.g., depression, posttraumatic stress disorder, anxiety) (73–75). The vagal functional mechanism began with activation and regulation of the HPA generating corticotropin-releasing-hormone (CRH), which coordinates the organism’s adapting stress reaction and physiological homeostasis (76).

Environmental stress and systemic proinflammatory cytokines cause hypothalamus secretion of corticotropin-releasing factor activating the HPA. The corticotropin-releasing factor is also involved in stimulus on the pituitary gland followed by adrenocorticotropic hormone secretion. This stimulation causes the release of cortisol, an immunosuppressive mediator and major stress hormone that affects many organs (67, 77). The combined activity of the vagus nerve and HPA allows for high interaction between the brain and gastrointestinal cells, more specifically immune cells, epithelial cells, enteric neurons, smooth muscle cells, interstitial cells of Cajal, and enterochromaffin cells, that practically are all modulated by the microbiota (67). Enterochromaffin cells, as part of enteroendocrine and neuroendocrine cells, are one of the most important influencers of bidirectional brain-gut communication. Behind the innervation produced by the vagus nerve on enterochromaffin-cell signaling to neuronal circuits, response to pain, background emotions, and immune-response modulation can seriously be changed. In the case of germ-free animal studies, it was observed that microbial colonization of the intestine induces normalization of HPA response, supporting the idea of the existence of a critical time interval that allows for neural plasticity to process input from gut microbiota (50).

## Neurological Disorders Directly Impacted by Changes in Microbiota

Gut microbiota communicate with the CNS, biosynthesize neurotransmitters, and influence neurological health status. They are also responsible for human behavior, mood status, and emotions (60, 78). The gut microbiota’s interplay with the CNS has a crucial impact on the appearance and progression of neurodegenerative disorders and tumors of the CNS in some cases (79). Besides, as there is a symbiotic relationship between gut microbiota and the host, their dynamic disruption interaction leads to profound effects on host health, including damage to the cortex (80, 81). Even though neurodegeneration occurs because of ENS malfunction, a multitude of clinical studies shows that patients experiencing neurodegenerative disorders encounter gut dysfunction and microbial dysbiosis (81, 82). In accordance with this, there is a strong correlation between gut dysbiosis and the incidence of neurodegenerative illnesses, but it is not entirely known yet if dysbiosis is induced or the result of neurodegeneration progression. Therefore, more clinical investigations are needed to understand the process completely.

Moderate to severe neurological-associated affections such as depression, anxiety, autism, Alzheimer’s disease, Parkinson’s disease, schizophrenia, multiple sclerosis, and epilepsy, are all considered to be linked to alterations in gut microbiota, which are further linked with genetics or environmental factors (e.g., dietary and lifestyle habits, antibiotic therapies, geographical region, etc.) (47, 51, 83). Last but not least and in line with the altered lifestyle and dietary habits that endanger the state of general health, and with repercussions on mental health, anorexia nervosa is classified as another debilitating psychiatric disorder having dramatic physiological and psychological effects (84). Anorexia nervosa is a severe compulsive eating disorder with high morbidity and mortality that is characterized by indefatigable self-starvation, and it is associated with reduced rates of recovery mainly because of raised gastrointestinal disequilibrium (85, 86). The following review explores the most common mental-associated disorders, considering the impact of intestinal imbalance and the severity of the illness that developed.

### Depression

Depression, more precisely, major depressive disorder (MDD), with severe psychological morbidity, has frequent fatal outcomes determined by several crucial functions, particularly regarding appetite, constant low mood, cognitive processes, psychomotor activity, and sleep (87, 88). With continuously increasing prevalence, depression plays an essential role in the global disease burden, affecting around 264 million people (89). Biological, environmental, and genetic factors and their reciprocal relationship are considered the main triggers of MDD. At the moment, MDD is usually treated with antidepressants (e.g., escitalopram, imipramine, fluoxetine, phenelzine, venlafaxine, desipramine, bupropion, and aripiprazole), which strengthens in the synaptic cleft the neurotransmitter densities and hinders the cognate transporters in the brain, like norepinephrine, and serotonin. The same transporters and receptors related to depression are also found in the gut, which are in close interaction or are influenced by the gut microbiota (58, 90–93). In addition, tricyclics, selective serotonin reuptake inhibitors, monoamine oxidase inhibitors, and novel compounds are the four main classes of antidepressants according to Cussotto et al. (94). In accordance with recent studies, the host microbiome should be prioritized as a major target in the development of new psychotropic drugs for the treatment of mental illnesses, as multiple antidepressant medications exhibit antimicrobial activity on representative strains of the human gut microbiota, especially on *Akkermansia muciniphila*, *Bifidobacterium animalis*, and *Bacteroides fragilis* (95, 96). In line with the research conducted by Macedo et al. (97), antidepressants have antimicrobial effects that may be related to the efficacy of drugs used to treat MDD; at the same time, some antimicrobials have neuroprotective and antidepressant effects. Therefore, the mechanisms of antidepressants in MDD treatment are extremely complex, and more research is required to make a stable conclusion (97). Microbiome alteration may be one of the primary triggers of peripheral immune response disruption in patients with depression (88). Nevertheless, a large percentage of the population is unresponsive to standard medications for depression (98). Although inflammatory reactions and excessive stress are involved in the sustainment of depression, the gut microbiota also play a major role in neuropsychology and pathway disequilibrium (99, 100). This interlinking connection among gut microbiota, IS, and stress matrix was evidenced in two recent reviews by Cheung et al. (91) and Cruz-Pereira et al. (100) who suggest that stress response triggers the fight-or-flight approach (through the HPA axis), and that together with all signaling factors generated by microbiota causes neuro-inflammatory processes that further induce depression.

Numerous studies prove that the brain-gut-microbiota axis represents an essential part in the evolution of depression, and comparing the gut microbiota of healthy individuals with those of individuals with depression, the latter is substantially altered (50, 101–104). A study by Jiang et al. (105) found that comparing the intestinal dysbiosis between MDD patients and healthy controls resulted in significant differences in the three main levels of phyla like increased Proteobacteria and Bacteroidetes, and decreased Firmicutes percentage. Additionally, *Bacteroides* level was significantly decreased in patients with MDD. Moreover, species of *Alistipes* genera and *Enterobacteriaceae* phylum were higher, which was also associated with several metabolic diseases (105). The same conclusion was drawn by Zheng et al. (106), who demonstrated that mice colonized with the gut microbiota of patients with MDD led to elevated depression-like demeanor. Here, no significant difference was found at the level of phyla for Firmicutes in patients with MDD and healthy individuals, only in some members within the phylum Firmicutes. Naseribafrouei et al. found an excessive proportion of Bacteroidales in patients with MDD and a restrained amount of Lachnospiraceae (107).

Evidence from clinical trials on patients with MDD (Supplementary Table 1) suggests that their intestinal microbiota are significantly different from those of healthy individuals. Even though these studies pointed out disturbances in gut microflora, precise discrepancies between patients diagnosed with MDD and healthy individuals are still in discussion, and further investigations are needed.

### Anxiety

Anxiety conditions are quite frequent among adult people, and generalized anxiety disorder (GAD) is among the most widespread and chronic forms diagnosed (108, 109). Worldwide, GAD affects around 4–6% of the total population, creating significant personal and financial burdens (110, 111). GAD is reflected by habitual negative thinking, swinging thoughts (i.e., concentration difficulties, rambling), irritability, sleep problems, muscle tension, and extreme or uncontrolled everyday anxieties produced for at least 6 months (112, 113). GAD has been deemed a chronic disease, since its duration can affect patients even for periods of over 12 years (114).

In anxiety situations, amygdala malfunction (HPS axis) is a characteristic trait that has the same vulnerability to environmental challenges as gut microbiota throughout a lifetime (115). Both amygdala and microbiota dysfunctions intersect with the onset of several psychiatric diseases (43). Gastrointestinal tract inflammation instigates the liberation of pro-inflammatory cytokines, and increase in tumor necrosis factor-alpha (TNF-α) and interleukin 6 (IL-6) cytokines is linked directly with anxiety-like manifestations (101). Meanwhile, serotonin has an essential role in gastrointestinal performance and gut-brain axis connection, and it has an important role as a neurotransmitter in cognition and mood. By perturbation of serotonin production (generated mainly in the digestive tract), mood and anxiety disorders can be triggered (66). In addition, anxiety-like episodes in humans are treated mostly with selective serotonin and norepinephrine reuptake inhibitor-based medications (e.g., fluoxetine, sertraline, paroxetine, citalopram, venlafaxine, and duloxetine), which affect the profiles of the gut microbiota (108, 116).

A study conducted on germ-free mice demonstrated that intestinal microbiota transferred from mice without maternal separation positively impacts intestinal dysbiosis and diminishes anxiolytic behavior (117). There are several other studies on animals outlining the relationship between anxiety and gut microbiota (118–120) but very few on individuals with GAD. The first study conducted on patients with GAD revealed diminished gut microbiota, encompassing alterations amid eight genera (121). Here, they observed an increased abundance of *Bacteroides* compared to the microbiota of healthy individuals, which can be related to anxiety-like disorders. Another study outcome was the low preponderance of SCFA-generating bacteria (*Sutterella*, *Faecalibacterium*, *Lachnospira*, *Eubacterium*, and *Butyricicoccus*) in patients with GAD. The small amount of *Faecalibacterium* strengthens the assumption of a previous study (105) regarding the positive outcome of psychiatric disorders of the increase of this genera in the gut microbiota (Supplementary Table 1). Several studies conducted on animal models and human patients reveal the beneficial effect of probiotics such as *Lactobacillus* and *Bifidobacterium* on anxiety (115, 122). Messaoudi et al. observed the beneficial anti-anxiety effect of probiotics composed of *Lactobacillus helveticus* R0052 and *Bifidobacterium longum* R0175 on both rats and humans (123).

### Bipolar Disorder

Bipolar disorder (BD) is a severe and recurrent neuropsychiatric disorder that affects more than 1% of individuals and is the 17th burden of disease worldwide after depression and anxiety disorders (124). Important drivers comprise fundamental brain alterations (chronobiology and neuroplasticity disruptions), pathophysiological reasons (environmental or genetic causes), or nitrosative and oxidative stress, calcium and neurotrophin signaling route, and cellular bioenergetics transformations (125). Patients affected by BD can experience either hyper-manic or depressive and manic conditions. BD is defined by periods of acute low mood, feelings of despair, intense unhappiness and disinterest in life (depressive) or high mood, extremely cheerful thinking, and low sleep requirements (manic). These symptoms lead to decreased life quality and are distinguished by functional or mental drawbacks and serious risk of fatality through suicide (44).

The bidirectional connection among the ever-changing gut microbiome, disposition, and cognitive disruption is supported by several studies (126). Compared with healthy individuals, patients diagnosed with BD display enhanced bacterial translocation markers that originated from the intestinal lumen, underpinning the elevated inflammation that possibly represents these conditions (127, 128). BD is also linked with metabolic disturbances and obesity, which also leads to complicated disease outcomes and worse prognosis (129, 130). Nowadays, patients diagnosed with BD are treated pharmacologically with mood stabilizers (mostly antidepressants) such as lithium, valproate, carbamazepine, lamotrigine, olanzapine, and fluoxetine, that exert modifications in the microbial community in the gut (44, 128).

A study on stool microbiomes proved that the microbiome of individuals with BD was significantly different from the microbiome of healthy individuals (131). An important finding was the small amount of the genus *Faecalibacterium*, an autochthonous gut bacterium that can be linked to diseases or depressed conditions. The same low amount of *Faecalibacterium* was found in a study by Painold et al. (125). A positive mental outcome could be achieved with an increase in *Faecalibacterium* levels (132) but with great care due to reported gastrointestinal disorders observed in subjects with BD (44). Of note, McIntyre et al. (133) reported a four-fold lower amount of Clostridiaceae in participants with BD than healthy controls (133). *Clostridiaceae* and *Collinsella*, are accountable for carbohydrate fermentation and SCFA generation (134), and *Clostridiaceae* are also considered important in gut barrier integrity sustainment. Painold et al. (125) also reported an increased level of *Coriobacteriaceae* that are connected to elevated cholesterol levels (135), and an increased amount of *Lactobacilli* is also considered an assisting factor in obesity concerning BD (125, 136). In patients diagnosed with both BD and MDD, low counts of *Bifidobacterium* due to low cortisol production are believed to play a negative role in stress response. On the other hand, an increased proportion of *Lactobacillus* might be advantageous for sleep disorders (92, 93) (Supplementary Table 1).

The frequency and severity of depression, anxiety, and bipolar disorders can be adjusted through diet. Several studies support the association between the Mediterranean diet, which is mostly based on plant foods, fresh fruits, olive oil, fish, reasonable amounts of dairy products, low amounts of red meat, and low to moderate amounts of wine, and reduction of depression in young and older adults (20, 137–140). On the other hand, the Western diet rich in processed or fried foods, refined grains, and sugary products is linked with increased levels of anxiety, depression, and bipolar behavior in women (137, 141).

### Autism

Autism spectrum disorder (ASD) is considered one of the most serious global neurodevelopmental conditions that have been associated with alterations in intestinal microbiota (6, 142). Based on recent publications, the prevalence of ASD in children and adolescents is increasing, with specific higher predominance in boys than in girls (49). According to an article from 2017, 1 in 68 children is affected by ASD in the United States (143), while in two publications from 2019 to 2020 it was reported that 1 in 59 individuals is diagnosed with ASD in the United States (144, 145). ASD is defined by deviant behavior, insufficiencies in communication and collective interactions, and lack in the ability to build relationships, which can range from relatively insignificant to severe or debilitating (143, 146).

In some studies, it is pointed out that ASD is caused by genetic factors, IS malfunction, inflammation, and other possible external factors. Gut microbiota are one of the most important factors that are directly involved in the severity of this disease. Alteration in gut microbial profile by drugs, antibiotic therapies, or inadequate nutrition is closely linked with abnormal emotional behavior and neurological malfunctioning triggering ASD development (142, 147, 148). The most frequent gastrointestinal symptoms associated with ASD, with a prevalence range of 23–70%, are abdominal pain, constipation, gaseousness, diarrhea, and flatulence (142, 149).

The pathogenesis of ASD is linked with an alteration in metabolism of an essential aromatic amino-acid, tryptophan (6, 150), which is induced by changes in the structure of gut microbiota (144, 151). Tryptophan is converted by intestinal microorganisms into biologic active molecules like indole and indole-derivatives, which are essential in maintaining neurological homeostasis (152–154). Therefore, increased levels of tryptophan and its derived metabolites in urinary excretion and their decreased levels in plasma are associated with a high rate of ASD development (155, 156). Moreover, an abnormal level of SCFAs (e.g., propionate) produced by commensal bacteria that accumulated in the brain is correlated with autistic symptoms in mice models and children diagnosed with ASD (7, 157).

Dysbiosis generated in microbial gut communities and in their metabolic products directly impacts the development of ASD in both animal models and children (158). In a meta-analysis conducted by Xu et al. (142) on 9 studies including 254 patients diagnosed with ASD, it was pointed out that children affected by autism present with decreased concentration in *Akkermansia*, *Bacteroides*, *Bifidobacterium*, and *Parabacteroides* communities, and increased concentration in *Faecalibacterium* community from total detected microorganisms compared to controls (142).

Some studies show that the maternal microbial luggage modulates later neurological development. For instance, breastfed infants for more than 6 months are associated with a diminished risk of ASD development (159), while infants fed from birth with breast milk through a bottle or formula-fed infants are predisposed to neurological and behavioral changes associated with ASD development (160). Besides, maternal lifestyle and habits like smoking, alcohol abuse, drug consumption, and HFD during pregnancy induce changes in the offspring’s microbiota and their later social behavior (148, 161–164) (Supplementary Table 1).

At present, there are no definitive and effective therapies for the treatment of ASD. Administration of supplements such as vitamins, pre- and pro-biotic formulations, and multi-minerals, and personalized diet and psycho-pharmacological and educational therapy are alternative methods for families that face this situation.

### Schizophrenia

Schizophrenia (SCZ) has been regarded as a destructive neuropsychiatric disease characterized by considerable diverse, multifaceted disorders; it affects about 21 million of the world’s population (<1%) (165, 166). According to Owen et al. (167), in SCZ, there are three main types of manifestation that can be characterized as “positive” symptoms (torn from reality with hallucinations, and delusions), “negative” symptoms (disabled incentive, declined free speech, and socially isolated), and intellectual disabilities (low performance, several mental disorders) (167). The life expectancy in this disorder is anticipated to be below 15 years compared to healthy people because of comorbid physical health issues and suicidal predispositions (168).

The main causes of SCZ are not clarified but are presumed to be complex, including environmental and genetic factors, generally with onset before full brain maturation (169, 170). As several neurotransmitters (GABA, norepinephrine, serotonin, and dopamine) are produced by microbes, especially as dopamine is essential in SCZ disease development and progression, it is appropriate to say that psychotic illnesses are regulated by the gut microbiota (171). In most neurodevelopmental disorders, as well as in SCZ, gut dysbiosis has a prominent role in affecting behavior (172), so gastrointestinal inflammations and decreased intestinal motility are related to SCZ progression (173, 174). Permanent stress related to SCZ and other mental diseases have a negative effect on microorganisms belonging to the gut microbiota (165). Also, individuals with SCZ often develop other metabolic syndromes like diabetes, obesity, hypertension, and cardiovascular disease because of antipsychotic treatment or essential metabolic deviations (175) (Supplementary Table 1). Patients diagnosed with SCZ who are treated with neurotransmitter-based medications (e.g., chlorpromazine, clozapine) having an antipsychotic effect show differences in gut microbiota profiles (167, 175).

To elucidate the relationship between gut dysbiosis and SCZ, several studies were conducted, especially on Chinese individuals, where the gut microbiota of patients with SCZ and healthy controls were compared (176). Zheng et al. (177) found significant alterations between the gut microbiota of patients and controls, and microbial composition was not as diversified (unusually low α-diversity count) as in healthy subjects. The abnormal microbial situation was mainly defined by the low count of Ruminococcaceae and Lachnospiraceae families, both from the Clostridiales order and essential in healthy gut preservation. Other fundamental differences observed were an enhanced count of bacterial families like Prevotellaceae, Veillonellaceae, Bacteroidaceae, and Coriobacteriaceae, compared to healthy controls (177). Shen et al. (178) also reported diminished levels of Clostridiales order (Lachnospiraceae and Alkaligenaceae) and increased levels of Proteobacteria (Prevotellaceae, Veillonellaceae, Lactobacillaceae, etc.) in patients with SCZ (178). Generally, there is a significant unconformity among studies on bacterial taxa, and an increase in the sample size of patients and controls could give a better overview of the relationship between dysbiosis and SCZ (176, 179, 180).

### Parkinson’s Disease

Parkinson’s disease (PD) is the second most common neurodegenerative disorder (affecting approximately 3 million people), exceeded by Alzheimer’s, and is the primary most ordinary movement disorder (181). It is a characteristic disease of the population over 60 years old and it expects a double incidence rate between 2005 and 2030 (182). PD results from low level of dopamine molecules, caused by dopaminergic neurons deterioration, which are located in the brain’s substantia nigra (183–185).

PD’s symptoms are categorized into motor symptoms such as bradykinesia, rigidity, resting tremor, and postural instability, and non-motor symptoms such as constipation, hyposmia, orthostatic hypotension, anxiety, insomnia, pain, and urogenital dysfunction (186–188). According to many clinical studies, and following post-mortem examinations, research studies have inferred that the first signs of PD appear in the ENS in the form of gastrointestinal abnormalities (constipation), usually several years before the onset of locomotor signs (181, 187). This notion is supported by the identification and accumulation of the amyloid protein α-synuclein in the gut from where it propagates to the brain through the vagus nerve (181). α-Synuclein protein overexpression is a toxic mediator in the pathology of PD (189). In addition, the pharmacological medication for patients with PD is mostly based on dopamine replacements, like 3,4-dihydroxy-L-phenylalanine, also known as levodopa (185, 186, 190).

Gut microbiota play a very important role in the evolution and symptoms of PD. This aspect was proved by a research study conducted by Scheperjans et al. (191), where fecal microbiomes of 72 patients with PD were compared with those of 72 control subjects (healthy participants). The final results showed that the patients with PD had a 77.6% lower percentage of Prevotellaceae compared to control patients’ microbiome and abundance of Enterobacteriaceae (191). Moreover, alterations in gut microbiota composition are associated with the severity of postural instability and gait difficulty (192).

According to recent investigations, diet is a key factor for symptom decrease and prevention of PD. A research study published by Perez-Pardo et al. (190) highlights that dietary intervention based on docosahexaenoic acid (DHA) and uridine might prevent motor and non-motor symptoms of PD and rotenone-induced motor and gastrointestinal dysfunctions in mice affected by PD (190). Agreeing with many research papers, epidemiological evidence of rotenone exposure may be a risk factor for developing PD, due to dopaminergic neurotoxicity expression (13, 193). More studies point out the possible relevance of the Mediterranean diet in decreasing the risk of PD. The Mediterranean diet implies fresh and unprocessed food consumption, such as food products rich in polyphenols, monounsaturated and polyunsaturated fatty acids, and, most significant in PD, high dietary fiber intake (25–30 g/day) (194, 195). A high amount of dietary fibers represents an energy source for SCFA-producing bacteria and, in this way, the Mediterranean diet ensures higher content of anti-inflammatory products like lactate and succinate (194). A randomized clinical trial (82 patients with PD) has shown that the Mediterranean diet considerably improved the total score of cognitive function in patients diagnosed with PD-dementia, such as visuospatial abilities, aspects of executive functions, short-term memory recall, language score, attention, concentration, working memory, orientation to time and place (195).

Nevertheless, more research is necessary for better understanding of the communication between the gut and the brain, focusing on the connection that exists from the gut microbiota to the pathophysiology of PD.

### Alzheimer’s Disease

Alzheimer’s disease (AD) is the most frequent cause of dementia in aging societies and results in the progressive and irreversible decline in cognitive functions. Intracellular accumulation of neurofibrillary tangles composed of hyper-phosphorylated τ-protein and extracellular plaques that consist of amyloid-β (Aβ) peptide defines the pathology of AD (196). Aβ deposits are thought to be associated with oxidative damage and neuroinflammation in the brain, leading to synapse loss and neuronal death. Its production and accumulation commonly begin around the age of 40 but may take more than 20 years to manifest as cognitive impairment; therefore, AD progression is too advanced for treatment once it becomes clinically obvious (197). This fact highlights the importance of preventing and delaying the onset of AD through dietary and lifestyle habits.

Neurodegenerative disorders usually reveal in advanced age, when gut microbiota composition is affected by diverse factors, such as poor diet, drug intake, and others. The onset of AD is linked to a physiological cascade that begins with increased permeability of the gut barrier and immune activation, in time leading to systemic inflammation that may further impair the blood-brain barrier and promote neuroinflammation, neural injury, and, ultimately, neurodegeneration (198). Age-related alterations in gut microbiota composition, namely, decreased diversity and stability, lead to the release of significant amounts of amyloids, LPS, and other microbial by-products into their surrounding environment. These secondary metabolites may be associated with a persistent inflammatory state of the gut mucosa that is connected to gut barrier breakdown. Absorption of these molecules affects signaling pathways related to the production of pro-inflammatory cytokines, some of which are related to the pathogenesis of AD (199). Anyway, medicinal treatments used by therapists to prevent and ameliorate the symptomatology in patients with AD are connected with metformin (23, 200, 201), a chemical compound with a metabolic mark especially in insulin resistance (201).

Clinical data referring to gut dysbiosis in AD revealed that increased abundance of pro-inflammatory gut microbiota taxon *Escherichia*/*Shigella* and decreased abundance of anti-inflammatory *Eubacteriumrectale* were possibly associated with a peripheral inflammatory state in elderly patients with cognitive impairment and brain amyloidosis (202). Gut microbiota alterations were also observed in fecal samples from age- and sex-matched individuals through genus-wide differences in bacterial abundance, namely, decreased Firmicutes and *Bifidobacterium* and increased *Bacteroides* in the microbiota of participants with AD (203). Interestingly, some associations are related to the Gram-negative bacteria’s outer membrane and are formed because of LPS and AD pathology. According to Zhan et al. (204), LPS promotes the formation of amyloid-like plaques in the rat brain, and further research on the human brain suggests the possibility that LPS in combination with other factors could cause AD neuropathology.

In conclusion, evidence from both animal and human studies supports the association between gut dysbiosis and microglia activation during AD development and promotes novel strategies for AD therapy by remodeling the gut microbiota (205). The most common non-invasive modulatory factor in this direction is a diversified diet that can ensure the necessary substrate for gut colonization predominantly by Firmicutes. Since genetic factors remain uncontrollable, AD development may be modifiable by the management of indirect risk factors, such as lifestyle habits. Some controllable risk factors were framed by Barnard et al. (200) in a dietary and lifestyle guideline for the prevention of AD and refers to, among others, physical exercise, sleep routine, and regular mental activity that promotes new learning. Scarce clinical evidence is available (Supplementary Table 1, on the efficacy of meditation for improving cognitive function, stress reduction, sleep, emotional regulation, and related physiological outcomes. Larger, more rigorous randomized controlled trials are needed to evaluate meditation as a therapeutic intervention (206). However, some findings suggest that meditation practice could reduce age-associated structural and functional brain changes in elderly expert meditators (207).

### Dementia

Dementia is a psychological disorder characterized by a group of symptoms, including memory illnesses, personality changes, thinking, and social ability decrease, and impaired reasoning. All these symptoms have physical, psychological, social, and economic impacts on patients, families, and societies. Worldwide, dementia presently affects 50 million elderly people, and the increase expected by 2,050 is approximately 130 million individuals (208).

Behavioral and psychological symptoms of dementia (neuropsychiatric symptoms) are represented by agitation, aberrant motor behavior, anxiety, elation, irritability, depression, apathy, disinhibition, delusions, hallucinations, and sleep or appetite changes (209). Many research studies confirm that the primary risk factor of dementia is neurodegeneration due to AD in older people, and around 60–70% of patients with AD end up developing dementia, followed by the second cause of dementia, cardiovascular and cerebrovascular diseases (including stroke, clinically silent brain infarcts, and cerebral microvascular lesions) (208, 210, 211). Beginning with childhood, some protective factors and mechanisms for preventing dementia can be included in an individual’s lifestyle. The first is education, followed by physical, cognitive, and social activities.

Randomized clinical trials (Supplementary Table 1) are made for the analysis and prevention of cognitive decline and dementia in elderly adults, aiming to reduce primary risk factors, such as unhealthy diet, alcohol abuse, smoking, diabetes mellitus, depression, genetic factors, and familial aggregation, hypertension, obesity, and) (208). Knowing that metabolic and inflammatory pathways have an essential role in neurodegeneration, microbiota control and modulation can be key to preventing dementia.

Decrease and increase in *Bacteroides* have direct implications in reduction or stimulation of risk factors of cognitive declines such as dementia (203, 212). *Lactobacillus*, *Bifidobacterium*, and *Streptococcus* are the most studied probiotic strains with multiple beneficial effects on human health such as stimulation of immune processes in the host, being a barrier to pathogenic organisms by adherence, reestablishment of microbiota, restoration of health, and production of metabolites with antimicrobial effects (212). In the context of dementia, it is believed that *Lactobacillus* and *Bifidobacterium* can improve the activity and release of neurotransmitters, being a protective factor against this particular illness especially by stimulating the production of acetylcholine, which is a metabolite closely associated with neurotransmitters in the central and peripheral nervous system affecting learning and memory processes (212–214).

A study conducted by Saji et al. (214) has demonstrated the relationship between gut microbiome-associated metabolites and dementia. In their study, fecal samples from two groups of patients were investigated; the group without dementia included 82 individuals and the group of patients with dementia included 25 individuals. The results show that the concentration of metabolites such as phenol, p-cresol, indole, and ammonia was higher in demented patients than in control individuals. Fecal ammonia was associated with the presence of dementia, because it represents a higher risk factor for cognitive impairment and AD (214).

There is extensive literature on mechanisms involved in gut microbiota modulation and dementia, a neurodegenerative disorder, but the association between probiotics and cognitive function improvement in humans affected by dementia needs more targeted research.

### Multiple Sclerosis

Multiple sclerosis (MS) is another neurologic-associated disorder that is triggered by inflammatory processes, which might be initiated through alterations in intestinal microbiota and malfunctions at the CNS level (6, 215). Worldwide, the prevalence of MS exceeds 2.3 million affected individuals (216, 217). MS is characterized by demyelination of nervous cells, which leads to neurodegeneration. The demyelination process is produced by the activity of immune cells such as B cells and autoreactive T lymphocytes with specificity for myelin antigens (216). The most common symptoms of MS following demyelinated lesions are tiredness, torpidity, lack of coordination, vertigo, loss of vision, muscular pain, dysfunctions of bladder and bowel, and depression (218, 219).

The gut microbiome stimulates and modulates the IS, and has, at the same time, a very close connection with the severity of MS (220). Biological molecules like neurotransmitters (GABA, histamine, serotonin, dopamine, etc.) and intestinal lymphocytes, cytokines, or chemokines excreted by intestinal microorganisms through sympathetic or parasympathetic ways of CNS influence the demyelination process of axons in the brain and spinal cord (221, 222). Moreover, the synthesis of abnormal gut microbial metabolites (SCFAs) interferes with the function of lymphocytes and the production of cytokines, which could further trigger the inflammatory process of demyelination and neurodegeneration (61, 222, 223). The gut dysbiosis of patients affected by MS is correlated with expanded intestinal permeability, microbial translocation, and local and systemic inflammation due to the large number of generated pro-inflammatory mediators (e.g., TNF-α), which decrease the expression of tight junction proteins, causing increase in intestinal barrier permeability (224). Medications dedicated to the treatment of MS symptomatology include immunosuppressive drugs such as teriflunomide, cyclophosphamide, mitoxantrone, rituximab, daclizumab, basiliximab, azathioprine, methotrexate, mycophenolatemofetil, beta-interferon, and glatiramer, which are correlated with modifications in the gut microbiome and immune transcriptome (225).

It was observed that microbial diversity from fecal samples of patients diagnosed with MS presents an increased level of Euryarchaeota and Verrucomicrobia phyla compared to healthy persons. Specifically, there were observed increases in *Methanobrevibacter* and *Akkermansia* and decreases in *Butyricimonas* species (221, 225). In other clinical studies involving patients with relapsing-remitting MS, reduction in *Parabacteroides distasonis* and *Prevotella copri*, and increase in *Dorea* and *Blautia*, the last two species connected with inflammatory diseases like Crohn’s disease (226), were noticed. In a study conducted by Cantarel et al. (227) on patients affected by MS and treated with glatiramer-acetate (an immunomodulator medication currently used for MS treatment), differences in microbial communities like Bacteroidaceae, Lactobacillaceae, and Clostridiales were noticed compared with untreated patients. The same study showed that after glatiramer-acetate-untreated patients with MS were supplemented with vitamin D, an increase in the *Akkermansia*, *Faecalibacterium*, and *Coprococcus* genera was noticed (227) (Supplementary Table 1).

Because MS is a very complex disorder implying multiple associated factors (environmental, genetics, diet, lifestyle), its treatment could approach gut microbiota’s modulation to improve the clinical results of patients diagnosed with MS. As Calvo-Barreiro et al. (228) highlight in their review article, gut microbiota’s marks might be used as potential pathogenic signatures and biomarkers for early detection of MS. At the same time, the intestinal microbiota could be much more explored for their immunomodulatory properties to treat MS (228).

### Epilepsy

Epilepsy is a widespread heterogeneous group of nervous system disorders with a worldwide prevalence of more than 70 million people (229, 230) and frequency in approximately 0.5–1% of children under 16 years of age (231). Epilepsy can be generated by several reasons like stroke, trauma, CNS infections, genetic mutations, cerebral malformations, and other undetermined causes (232). The main characteristics of epilepsy are recurrent seizures, neuro-inflammation, cell death, neurogenesis, and hyper-synchronized outbursts of unnatural network activities in the brain (233). Epilepsy, at first, was treated with antiepileptic drugs. Still, 25–30% of individuals usually fail in this type of therapy (drug-resistant epilepsy), and they receive alternative treatments like vagus nerve stimulation and brain surgery (234).

In drug-resistant epilepsy (example of antiepileptic drugs: zonisamide and lamotrigine), the gut-microbiota of patients have an altered composition and enhanced and multiple rare bacteria, like the phylum of Firmicutes and Verrucomicrobia, and reduced commensal bacteria (229, 232). In 30 patients with idiopathic focal epilepsy compared to healthy controls, the Proteobacteria phylum, including *Neisseria*, *Lautropia*, *Delftia*, *Campylobacter*, and *Haemophilus*, was found in higher quantity (235). In the same study by Şafak et al. (235), the Fusobacteria phylum was another species of bacteria found in epileptic patients and not found in healthy controls. Xie et al. (236) reported the same increased Proteobacteria phylum. These results indicate that inflammation and autoimmune mechanisms are responsible for the generation of epilepsy. A preventive element in epilepsy could be given by *Lactobacilli* and *Bifidobacteria*, together with rehabilitation of the gut microbiota (229). An ancient although only recently popular treatment for epilepsy is proposed through the improvement of gut dysbiosis, with a low-carbohydrate and high-fat diet, namely, ketogenic diet (KD) (237). Gut microbiota components are modified by implementation of KD and have a beneficial effect on metabolic, neuropsychiatric, and neurodegenerative manifestations, as demonstrated in several studies on animals and humans (238).

Two mouse models by Olson et al. (239) demonstrated the antiepileptic effect of KD. After the transfer of microbiota from KD-fed mice to control diet-fed mice, seizure protection was provided. In the KD-fed mice, although the gut microbiota had diminished abundance, enhancement in *Parabacteroides merdae*, and *Akkermansia muciniphila* could be observed (239). They also confirmed the anti-seizure effect of these two bacteria administered simultaneously in the mice. A recent exploratory study investigated the outcome of KD implementation in 20 children with refractory epilepsy after 6 months (240). Ten patients had under 50% reduction in seizure frequency, and the other 10 had higher than 50% reduction or were seizure-free, and they also presented better electroencephalogram results. The composition of gut microbiota was represented by increased Bacteriodetes and decreased Actinobacteria and Firmicutes phyla. Patients with low seizure reduction had a high number of *Alistipes*, *Clostridiales*, *Rikenellaceae*, *Ruminococcaceae*, and *Lachnospiraceae* (240) (Supplementary Table 1).

Another method for reducing seizure intensity, increasing GABA activity, and improving cognitive performance is the administration of probiotic bacteria, as displayed in a study on pentylenetetrazole-stimulated rats (241). Overall, although future research is still needed, the importance of gut microbiota in the modulation of seizure predisposition in animals and human epileptic individuals is demonstrated.

## Probiotic Formulations as a Potential Therapy for Neurological and Psychiatric-Associated Disorders

A considerable number of publications (reviews, original articles, and clinical trials) have appeared in the last decade related to the administration of prebiotic and probiotic formulations in the therapy of mental-associated disorders. In this regard, one important aspect is presented in two publications from 2013 (242, 243) where probiotics or prebiotics that directly influence gut microbes and brain relationships received the generic name of “psychobiotics.” The term was used further by researchers to define beneficial microorganisms and/or bacteria-derived compounds that precisely impact mental health status and can be administered as therapy in the prevention and treatment of neurological and psychiatric affections (137, 244–250). The main role of psychobiotics is to exert anxiolytic and antidepressant effects characterized by modifications in emotional, cognitive, systemic, and neural parameters (245). Lately, several meta-analyses conducted on the influence of psychobiotics on different mental disorders support the beneficial effect of these formulations as non-invasive therapy for mental-associated illnesses. For example, in a meta-analyses from 2020 conducted by Vaghef-Mehrabany et al. on the effects of psychobiotics on MDD, it is outlined that probiotic strains may have a positive impact on depressive symptoms (7 of 32 articles that met study criteria reported significant antidepressant effects on MDD) (251). Other clinical studies and meta-analyses also support the positive effect of probiotic strains such as *Lactobacillus rhamnosus* (JB-1) in terms of reducing stress-related behavior and corticosterone release in animal trials (252). Moreover, another meta-analysis highlighted that the probiotic strain of *Bifidobacterium breve* (CCFM1025) has shown promising results in attenuating both psychiatric and gastrointestinal abnormalities in patients diagnosed with MDD (253).

The well-known probiotic strains of *Lactobacilli* and *Bifidobacteria* are directly involved in the gut and mental homeostasis, especially because of their external structure that does not comprise pro-inflammatory LPS chains like the pathogenic strains of *Salmonella* or *Escherichiacoli* (243). *Lactobacilli* and *Bifidobacteria* are categorized as psychobiotics for their psychotropic properties in terms of behavior improvement in depressed and anxious individuals (243, 254, 255). In a study conducted by Liu et al. (254) on adult mice with and without induced stress in early life, it was observed that *Lactobacillus plantarum* strain PS128 reduces depression and anxiety-like behavior in tested animals. Moreover, *L. plantarum* PS128 increased the level of dopamine in the prefrontal cortical area of early life-stressed mice and increased serotonin levels in adult mice without induced stress (254). In a more recent publication, Liu et al. (256) treated stressed rats with *Lactobacillus fermentum* strain PS150 and investigated the serotonergic pathway. In this study, they observed that the intake of *L. fermentum* PS150 lowered the rats’ cognitive deficits. Also, the psychotropic effects of strain PS150 were demonstrated by a reduction in neurodegeneration and prevention of stress-mediated decrease in serotonin (256). In another study conducted on mice that were treated with a probiotic formulation that consisted of *Lactobacillus helveticus* R0052 and *Bifidobacterium longum* R0175, regeneration of tight-junction barrier integrity, reduction in HPA axis and ANS activities, and protection of the intestinal barrier were observed (105, 257).

Studies conducted on human hosts support the prophylactic effects of probiotics in terms of neurological development and their crucial impact on early life. In a clinical study conducted by Romeo et al. (258) on preterm infants, it was proved that strains of *Lactobacillus (L. rhamnosus* ATCC 53103 and *L. reuteri* ATCC 55730) administered in concentrations higher than 10^8^ CFU/ml for 6 weeks exerted neuroprotective effects that were visible through the Hammersmith infant neurological examination carried out at the age of 1 year (258). Moreover, behavioral improvement was noticed in 3–16-year-old children diagnosed with ASD and who were administered *L. plantarum* strain WCFS1 (daily dose concentration of 10^10^ CFU/ml) for 3 weeks. Children treated with probiotics showed progress in social behaviors, lower anxiety level, and fewer communication problems (259). In another cohort study comprising ASD-diagnosed children who were 4–10 years old and treated orally for 2 months with the probiotic strain of *Lactobacillus acidophilus* Rosell-11, it was noticed that their concentration ability was much more improved (260).

Probiotics were proved to have positive effects on reactivity to sad mood in adult humans by helping in the reduction of negative thoughts. Steenbergen et al. (122) administered a multispecies probiotic food-supplement consisting of *Bifidobacterium bifidum* W23, *Bifidobacterium lactis* W52, *L. acidophilus* W37, *Lactobacillus brevis* W63, *Lactobacillus casei* W56, *Lactobacillus salivarius* W24, and *Lactococcuslactis* (W19 and W58) to 20 healthy young adults for 4 weeks. At the end of the trial, using a revised Leiden index of depression sensitivity scale, a significant decrease in cognitive reactivity to sad disposition was observed (122). Another study investigated the favorable effect of probiotics on anxiety-related conditions and revealed optimistic results with reduced anxiety manifestations and improved social interaction in treated patients (261). In a clinical study performed by Barichella et al., it was observed that the administration of probiotics to patients suffering from severe constipation associated with PD improves substantially the patients’ bowel movement (262). Moreover, probiotics intake showed progress in cognitive function and metabolic status improvement, creating new preventive and therapeutic options for AD (197, 198, 263).

Probiotics as psychobiotics have a great potential in maintaining both gastrointestinal and neurological homeostasis. More studies must be conducted in the context of mental-associated affections on correlation with the health status of treated patients, age range, single- and multi-probiotic formulations, dosage, and time of administration. Prebiotics and probiotics administered as food supplements maintain the host’s general health status because of their resistance through the gastrointestinal tract, their good adhesivity to the intestinal mucosa, and their specific metabolites delivered in systemic circulation (264–266). Prebiotics, in particular, are non-viable food components that act indirectly by selectively stimulating the growth and activities of beneficial microorganisms in the gastrointestinal system, resulting in beneficial effects on the host, such as gastrointestinal, central nervous, immune, and cardiovascular system protection (255, 267). Few clinical research studies have investigated the neuropsychiatric effects of prebiotic formulations. However, Kazemi et al. (267) conducted a clinical trial using galactooligosaccharides as a prebiotic substrate to evaluate the diminution of depressive symptoms. Also, short-chain fructooligosaccharide prebiotics were used to evaluate the effects on patients with anxiety (268), fructooligosaccharides together with galactooligosaccharides to explore the effects on healthy volunteers’ stress hormone, cortisol, and emotional processing ability (269), and oligofructose-enriched inulin to investigate wellbeing, mood, and cognitive performance (270). Although the studies conducted found no significant effects of prebiotic formulation on neuropsychiatric illnesses, further randomized clinical trials are clearly required to assess the therapeutic potential of prebiotic formulations.

Pre- and probiotic formulations used as food supplements and at the same time used as psychobiotics must follow legislation quality requirements (given by World Health Organization), as they should contain specific microbial strains that must be sufficiently characterized, safe for the intended usage, should be supported by human clinical trials with positive outcomes designed in accordance with scientific standards or recommendations of local/national authorities, and, last but not least, must be viable and at an efficacious dose during their storage (69, 271). Moreover, in European Union countries, food supplements like pre-, pro-, and synbiotic preparations and novel foods containing these elements must follow the rules given by the European Food Safety Authority (EFSA), in particular regulation (EC) no, 1924/2006, and should be further authorized by the European Commission (272, 273).

**FIGURE 2:**
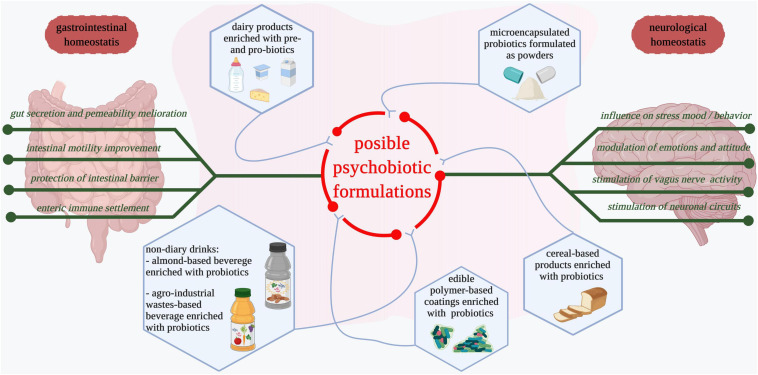
Possible formulations of psychobiotics. Dairy products such as yogurt, milk, and cheese fortified with probiotic strains are among the most consumed food products that have a positive influence on the gastrointestinal tract and have possible beneficial implications in mental health (274). Microencapsulated probiotics and those formulated as powders are commercially available and administered as supplements after antibiotic treatments (275, 276). Cereal-based products are used as vehicles for probiotics delivery (274). Different edible biopolymer-based coatings with entrapped probiotics are developed to facilitate both probiotics consumption and reduction of non-degradable packaging materials (277, 278). Fruits, vegetables, and agro-industrial waste-derived beverages enriched with probiotics are developed for easier probiotic and bioactive compound delivery (264, 279). Edible products supplemented with probiotics maintain gastrointestinal homeostasis and influence neurological health. Deeper studies that consider the impact of probiotic food products as psychobiotics in the prevention and management of mental-associated diseases are needed.

Figure 2 illustrates several types of pre- and pro-biotic preparations or novel foods that might be considered possible psychobiotic formulations and could be investigated in-depth considering their influence on mental-associated illnesses.

## Concluding Remarks

The gut microbiota shape the state of neurological and psychiatric health, and any type of imbalance in gut microbial communities or production of metabolites may be associated with imbalances at the CNS level resulting in the appearance of a disease.

This article presented various research studies, experiments on animal models, and clinical trials that highlighted the bidirectional communication between the gut and the brain through the microbiome-gut-brain axis in the pathogenesis of common neurodegenerative diseases such as depression diseases, anxiety, bipolar disorder, autism, schizophrenia, Parkinson’s disease, Alzheimer’s disease, dementia, multiple sclerosis, and epilepsy. Focusing on the bidirectional communication, gut-brain and brain-gut axis, we observed that the primary component responsible for information transfer is the vagus nerve, which has an important role in modulation of inflammation, maintenance of intestinal homeostasis, and regulation of food intake, satiety, and energy homeostasis. Moreover, the vagus nerve plays a significant role in the pathogenesis of psychiatric disorders as well as other stress-induced and inflammatory diseases.

In all investigated illnesses, it was observed that probiotics as psychobiotics play an important role in maintaining neuropsychiatric homeostasis. As a future perspective, more focus must be put on research on psychobiotics’ impact on health status in relation to patients’ age range, health issues, and genetic background, and on single- and multi-probiotic formulations, dosage, and time of administration.

## Author Contributions

LM and D-CV designed and outlined the review structure. LM, KS, B-ET, and S-AN carried out the literature search and wrote the first draft. LM and S-AN adapted and drafted the graphical abstract, Figures 1, 2, and Supplementary Table 1. LM contributed to the background, autism, multiple sclerosis, and probiotics formulations as a potential therapy for neurological and psychiatric-associated disorders sections. KS and S-AN constructed and described the gut-brain and brain-gut axis, a bidirectional communication section. KS contributed to the gut microbiota and neuropsychiatric status modulated by extrinsic and intrinsic factors and Alzheimer’s disease section. S-AN contributed to the Parkinson’s disease and dementia sections. B-ET contributed to the depression, anxiety, bipolar diseases, and epilepsy sections. D-CV revised the manuscript including the Figures’ structure. All authors contributed to the article and approved the submitted version.

## Conflict of Interest

The authors declare that the research was conducted in the absence of any commercial or financial relationships that could be construed as a potential conflict of interest.

## Publisher’s Note

All claims expressed in this article are solely those of the authors and do not necessarily represent those of their affiliated organizations, or those of the publisher, the editors and the reviewers. Any product that may be evaluated in this article, or claim that may be made by its manufacturer, is not guaranteed or endorsed by the publisher.

## Abbreviations

A β: amyloid-beta
ACTH: adrenocorticotropic hormone
AD: Alzheimer’s disease
ANS: autonomic nervous system
ASD: autism spectrum disorder
BD: bipolar disorder
CFU: colony-forming unit
CNS: central nervous system
CRH: corticotropin-releasing hormone
CVS: cyclic vomiting syndrome
ENS: enteric nervous system
ES: endocrine system
GABA: gamma-aminobutyric acid
GAD: generalized anxiety disorder
GALT: gut-associated lymphoid tissue
GRD: gastroesophageal reflux disease
HFD: high-fat diet
HPA: hypothalamic-pituitary-adrenal axis
IBS: irritable bowel syndrome
KD: ketogenic diet
IL-6: interleukin 6
IS: immune system
LPS: lipopolysaccharide
MDD: major depressive disorder
MS: multiple sclerosis
PD: Parkinson’s disease
SCFAs: short-chain fatty acids
SCZ: schizophrenia
TNF- α: tumor necrosis factor-alpha.
