# Supplementary material for: Guts Imbalance Imbalances the Brain: A Review of Gut Microbiota Association With Neurological and Psychiatric Disorders
Source: Front Med (Lausanne). 2022 Mar 31;9:813204. doi: 10.3389/fmed.2022.813204 (PMC9009523; doi:10.3389/fmed.2022.813204)
Supplement: Supplementary file 1 [file Table_1.docx]

**Table 1. Alterations in gut microbiota are closely linked with the evolution of some neuropsychiatric-associated disorders.**

| **Neurological-associated affections** | **Type of study** | **ENS associated symptoms** | **CNS associated symptoms** | **Gut-dysbiosis (genus)** | **Main metabolites associated with the illness** | **Key results of the study** | **References** |
| --- | --- | --- | --- | --- | --- | --- | --- |
| **Depression *(*MDD*)*** | Cohort:  1.054 individuals | Decrease of appetite, abdominal discomfort | Disruption of sleep, low psychomotor activity, cognition and constant low mood | ↓*Faecalibacterium*  ↓*Coprococcus*  ↓*Dialister*  ↓*Bacteroides* enterotype 1  ↓*Prevotella*  ↑*Bacteroides* enterotype 2 | ↓ butyrate  ↓δ-aminobutyric acid | - butyrate-producing *Faecalibacterium* and *Coprococcus* bacteria - associated with higher quality of life indicators  - *Coprococcus* and *Dialister* - depleted in MDD individuals | [99] |
|  | Cohort: 76 individuals (29-active MDD, 17-responded MDD, 30-healthy controls) |  |  | ↓*Bacteroides*  ↓*Faecalibacterium*  ↑*Alistipes* | -*data not shown* | - increased fecal bacterial α -diversity in active-MDD patients versus control group, but not in responded-MDD individuals  - Bacteroidetes, Proteobacteria, and Actinobacteria - strongly increased, Firmicutes- significantly reduced in active-MDD and responded-MDD groups | [105] |
|  | Cohort: 55 individuals (37- MDD patients, 18-non-depressed controls) |  |  | ↑*Alistipes*  ↑*Bacteroides*  ↑*Oscillibacter* | ↑valeric acid | - correlations between MDD and fecal microbiota was found: opposite directions even for closely related Operational Taxonomic Units, but were still associated with certain higher order phylogroups  - *Oscillibacter* produced valeric acid as the main metabolite; A*listipes* – associated with induced stress in mice | [107] |
|  | Cohort: 100 individuals (43 MDD patients, 57 healthy controls) |  |  | ↓*Bifidobacterium*  ↓*Lactobacillus* | -*data not shown* | -individuals whose bacterial counts below the optimal cut-off point were significantly more common in the MDD patients  -using the same cut-off points, was observed an association between the bacterial counts and IBS. Frequency of fermented milk consumption - associated with higher *Bifidobacterium* counts in MDD patients | [93] |
| **Anxiety**  ***(*GAD*)*** | Cross-sectional study: 76 individuals (40 GAD patients, 36 healthy controls) | Lack of appetite, abdominal discomfort, gastrointestinal tract inflammation | Irritability, sleep problems, muscle tension, concentration difficulties, rambling | ↓*Faecalibacterium*  ↓*Clostridium*  ↓*Sutterella*  ↓*Lachnospira*  ↓*Butyricicoccus*  ↑*Bacteroides* ↑*Fusobacterium*  ↑*Ruminococcus* | ↓SCFA | - markedly decreased microbial richness and diversity, distinct metagenomic composition with reduced SCFA-producing bacteria, overgrowth of *Escherichia*-S*higella*, *Fusobacterium, Ruminococcus gnavus*  - decreased abundances of *Faecalibacterium*, *Eubacterium rectale*, *Lachnospira*, *Butyricicoccus,* *Sutterella* in GAD patients - associated with gut barrier dysfunction | [121] |
|  | Cohort: 60 individuals (36 active-GAD patients, 24 healthy controls) |  |  | ↓*Prevotella*  ↓*Succinivibrio*  ↓*Mitsuokella*  ↓*Subdoligranulum*  ↓*Ruminococcus*  ↓*Eubacterium*  ↓*Dialister*  ↓*Subdoligranulum*  ↓*Megamonas*  ↓*Agathobacter*  ↓*Clostridium*  ↓*Coprococcus*  ↓*Acinetobacter*  ↑*Tyzzerella*  ↑*Hungatella*  ↑*Burkholderia*  ↑*Bacteroides*  ↑*Shigella*  ↑*Escherichia* | -*data not shown* | - *Bacteroides* - more highly in active-GAD patients, positively associated with GAD severity  - decreased abundance of Bacteroidaceae - associated with metabolic disorders like diabetes and obesity, and with effects on mood  - significant differences in gut-microbiota composition between GAD patients and healthy controls;  - microbiota profiles are affected by anti-anxiety medication, which in turn affect the severity of clinical symptoms. | [116] |
| **Bipolar disorders (BD)** | Longitudinal study: 179 individuals (115 BD patients, 64 control subjects) | Gastrointestinal tract inflammation | Acute low mood, feelings of despair, intense unhappiness, disinterest in life, high mood, extremely cheerful thinking, and low sleep requirements (manic) | ↓*Faecalibacterium*  ↓*Prevotella*  ↓*Roseburia*  ↓*Akkermansia*  ↓*Phascolarctobacterium*  ↓*Anaerostipes*  ↓*Parasutterella*  ↑*Bacteroides*  ↑*Alistipes*  ↑*Bifidobacterium* ↑*Parabacteroides*  ↑*Blautia*  ↑*Collinsella*  ↑*Lachnospira*  ↑*Acidaminococcus* | ↓ butyrate | - *Faecalibacterium -* associated with bipolar illness, negatively associated with self-reported burden of disease in BD individuals - independent sex, age, body mass index.  - therapeutically increasing *Faecalibacterium* in BD patients – associated with reduction of disease burden | [131] |
|  | Cross-sectional study: 42 individuals (32 BD patients, 10 healthy controls) |  |  | ↓*Faecalibacterium*  ↓*Ruminococcus*  ↓*Clostridium*  ↓Roseburia  ↓*Lactobacillus*  ↑Coriobacterium | ↕ tryptophan | - negative correlation between α- diversity and illness duration in BD  - bacterial clades associated with inflammatory status, serum lipids, tryptophan, depressive symptoms, oxidative stress, anthropometrics and metabolic syndrome in individuals with BD  - metabolic syndrome and low-grade inflammation - important trademarks for BD associated with gut microbiota. | [125] |
|  | Cross-sectional study: 229 individuals (113 BD patients, 39 unaffected first-degree relatives, 77 healthy controls) |  |  | ↑*Flavonifractor* | -*data not shown* | - gut microbiota community membership of BD patients differed from healthy individuals; community membership of unaffected first-degree relatives did not differ  -*Flavonifractor* – associated with oxidative stress and inflammation, and with BD. | [280] |
| **Autism (ASD)** | Cohort: 80 individuals (40 ASD subjects, 40 neurotypical controls) | Gastrointestinal tract inflammation, abdominal pain, constipation, gaseousness, diarrhea, and flatulence | Deviant behavior, insufficiencies in communication and collective interactions, inability to build interpersonal relationships | *↓Alistipes*  *↓Bilophila*  *↓Dialister ↓Parabacteroides ↓Veillonella*  *↓Prevotella*  ↑*Collinsella*  *↑Corynebacterium*  *↑Dorea*  *↑Lactobacillus*  *↑Escherichia*  *↑Shigella*  *↑Clostridium*  *↑Candida* | *-data not shown* | - altered intestinal microbial community (bacterial and fungal level) associated with ASD - not depending by the constipation status of autistic individuals but rather by the autistic disorder itself. | [281] |
|  | Cohort: 41 individuals (35 ASD subjects, 6 neurotypical controls) |  |  | *↓Veillonella ↓Streptococcus*  *↓Escherichia*  *↓Clostridium*  *↑Sutterella ↑Odoribacter ↑Butyricimonas* | *↓*butyrate  *↓*lactate  *↑*propionate  *↑*succinate  *↑*acetate | - butyrate and lactate producers - less abundant in the ASD group. - ASD - positively correlated with periodontal; negatively related to type 1 diabetes  - microbe-based disease analysis- able to predict connections between ASD and other diseases | [282] |
|  | Cohort: 29 individuals (10 ASD subjects, 9 siblings, 10 healthy subjects) |  |  | *↓Bacteroides*  *↑*Lactobacillus  *↑Desulfovibrio*  *↑Clostridium* | *-data not shown* | - frequency and severity of gastrointestinal symptoms in ASD children and in their siblings are higher than in control groups | [283] |
| **Schizophrenia**  **(SCZ)** | Cross-sectional study: 117 individuals (64 SCZ patients, 53 healthy controls) | Constipation, bowel obstruction, decreased intestinal motility | Hallucinations, delusions, disabled incentive, declined free speech, and socially isolated | *↓Blautia*  *↓Coprococcus ↓Roseburia*  *↑Succinivibrio ↑Megasphaera ↑Collinsella ↑Clostridium ↑Klebsiella*  *↑Methanobrevibacter* | ↓SCFA | - several metabolic pathways differed significantly between controls and SCZ patients, including vitamin B6 and fatty acids profile  - difference of gut microbiota between SCZ patients and controls | [178] |
|  | Cohort: 171 individuals (90 SCZ patients, 81 healthy controls) |  |  | *↑Lactobacillus ↑Enterococcus ↑Alkaliphilus ↑Cronobacter ↑Veillonella ↑Bifidobacterium ↑Dialister ↑Streptococcus*  *↑Bacteroides* | ↓propionate  ↓butyrate↓isovaleric acid)  *↓*tryptophan *↑*glutamate  *↓*δ-aminobutyric acid  *↓*nitric oxide  *↑*acetate  *↑*kynurenic acid | - greater α- diversity and higher β- diversity in SCZ patients, compared with healthy controls  -functional potentials associated with SCZ include differences in SCFAs synthesis, tryptophan metabolism, and synthesis/degradation of neurotransmitters | [172] |
|  | Cohort: 162 individuals (82 SCZ patients, 80 normal controls) |  |  | *↓Adlercreutzia*  *↓Anaerostipes*  *↓Ruminococcus*  *↓Faecalibacterium*  *↕Collinsella*  *↕Lactobacillus*  *↕Succinivibrio*  *↕Mogibacterium*  *↕Corynebacterium*  *↑Ruminococcus*  *↑Eubacterium* | *↓*polyketide sugar units  *↓*valine  *↓*leucine *↓*isoleucine *↓*pantothenated *↓*phenylpropanoid  *↑*ascorbate *↑*aldarate *↑*nucleotide *↑*propanoate | - α- diversity showed no significant differences between normal and SCZ groups; β- diversity revealed significant community-level separation in microbiome composition between the two groups  - *Succinvibrio* and *Corynebacterium -* associated with symptoms severity (new biomarkers for SCZ diagnosis) | [284] |
| **Parkinson's disease (PD)** | Cohort: 144 individuals (72 PD patients, 72 control subjects) | Constipation, hyposmia, pain, and urogenital dysfunction | Bradykinesia, rigidity, resting tremor, postural instability, anxiety, insomnia | *↓Prevotella* | *- data not shown* | - relative abundance of Enterobacteriaceae - positively associated with the severity of postural instability (motor phenotype) and gait difficulty | [191] |
|  | Cohort: 120 individuals (75 PD patients, 45 age-matched controls) |  |  | ↓*Lachnospira*  ↓*Fecalibacterium*  *↑Bifidobacterium*  *↑Clostridium*  *↑Bacteroides*  *↑Veillonella*  *↑Desulfovibrio*  *↑Paraprevotella*  *↑Escherichia*  *↑Enterococcus* | *- data not shown* | - significant increases in the abundance of four bacterial families  - significant decreases in the abundance of seventeen bacterial families in PD patients  - it was identified association between specific taxa and different PD motor phenotypes | [285] |
|  | Cohort: 115 individuals (64 PD patients, 51 healthy controls) |  |  | *↓Lachnospira*  *↓Butyrivibrio ↓Pseudobutyrivibrio*  *↓Coprococcus*  *↓Bacteroides*  *↓Blautia*  *↓Roseburia*  *↓Brevibacterium*  *↓Dolichospermum*  *↓Odoribacter*  *↑Akkermansia*  *↑Escherichia ↑Bifidobacterium ↑Streptococcus ↑Clostridium ↑Serratia ↑Veillonella ↑Prosthecobacter ↑Enterobacter ↑Slackia*  *↑*Desulfovibrio | *↓*linoleic acid  *↓*oleic acid *↓*nicotinic acid  *↓*glutamic acid *↓*pantothenic acid *↓*pyroglutamic acid  *↓*succinic acid  *↓*propionate  *↓*butyrate  *↓*sebacic acid  *↑*acetate  *↑*cadaverine *↑*ethanolamine *↑*hydroxypropionic acid  *↑*isoleucine  *↑*leucine *↑*phenylalanine  *↑*thymine | - microbiota modifications are correlated with numerous fecal metabolites  - PD - associated with gut dysregulation involving a synergistic relationship between gut microbes and several bacterial metabolites favoring altered homeostasis  - a reduction of SCFAs-producing bacteria influenced the shape of the metabolomics profile, affecting several metabolites with potential protective effects in the PD group | [82] |
| **Alzheimer’s disease (AD)** | Cohort: 50 individuals (25 AD patients, 25 age- and sex-matched control participants) | Symptoms associated with Irritable Bowel Syndrome | Synapse loss and neuronal death, psychological symptoms of forgetting, decline in the ability to perform activities of daily living | *↓Adlercreutzia*  *↓Bifidobacterium*  *↓Clostridium*  *↓Dialister ↓Turicibacter*  *↑Bilophila*  *↑Blautia ↑Phascolarctobacterium*  *↑Gemella*  *↑Bacteroides*  *↑Alistipes* | *- data not shown* | - gut microbiota of AD participants has decreased microbial diversity and is compositionally distinct from control age- and sex-matched individuals  - decreased Firmicutes, increased Bacteroidetes, and decreased *Bifidobacterium* - in AD participants’ microbiota  - correlations between levels of differentially abundant genera and cerebrospinal fluid biomarkers of AD individuals | [203] |
|  | Cohort: 17 individuals (11 AD patients, 6 control participants) |  |  | *↓Saccharomyces ↓Candida*  *↓*Meyerozyma  *↑Pichia*  *↑Cyberlindnera*  *↑Aspergillus*  *↑Cladosporium*  *↑Thermomyces*  *↑*Mrakia  *↑Botrytis ↑Kazachstania, ↑Phaeoacremonium*  *↑Cladosporium* | *↓*lactate  ↓acetate  ↓propionate ↓butyrate | - AD patients have higher proportion Sclerotiniaceae, Phaffomyceteceae, Trichocomaceae, Cystofilobasidiaceae, Togniniaceae  - Specific fungal taxa exhibit distinct correlation arrays with AD markers and gut bacteria in subjects with versus without AD | [286] |
| **Dementia** | Cohort: 17 individuals (25 patients with dementia, 82 participants without dementia) | Appetite changes, abdominal discomfort | Agitation, aberrant motor behavior, anxiety, elation, irritability, depression, apathy, disinhibition, delusions, hallucinations, and sleep | *↓Bacteroides*  *↓Prevotella* | *↓*succinic acid  *↓*lactic acid  *↓*n-valeric acid  ↕formic acid  *↑*acetic acid *↑*propionic acid  *↑*iso-butyric acid  *↑*butyric acid  *↑*iso-valeric acid  *↑*ammonia *↑*phenol  *↑*p-cresol  *↑*indol | - strong relationship between gut microbiome-associated metabolites such as ammonia and dementia, independent of traditional risk factors and gut microbiome  - fecal lactic acid concentration - inversely associated with dementia presence | [214] |
|  | Cross-sectional pilot study: 43 individuals (25 patients with dementia, 18 controls without cognitive impairment) |  |  | *↓Lachnospira*  *↓Eubacterium*  *↓Agathobacter*  *↑Clostridium*  *↑Streptococcus*  *↑Anaerostipes*  *↑Bacteroides*  *↑Faecalibacterium*  *↑Eisenbergiella*  *↑Ruminococcus*  *↑Lachnoclostridium* | *- data not shown* | - α- diversity did not change significantly with increasing degree of dementia  - dementia - associated with dysbiosis characterized by differences in β- diversity and changes in taxonomic composition  - increased gut permeability – associated with increased serum diamine oxidase levels and systemic inflammation; confirmed by increased soluble cluster of differentiation 14 levels  - reduced *Lachnospiraceae* NK4A136 - potential butyrate producer | [211] |
| **Multiple sclerosis (MS)** | Cohort: 103 individuals (60 MS patients, 43 healthy controls) | Systemic  inflammation,  dysfunctions of bladder and bowel | Tiredness, torpidity, coordination lack, vertigo, loss of vision, muscular pain, and depression | *↓Butyricimonas*  *↓Sarcina*  *↓Collinsella*  *↓Slackia*  *↓Prevotella*  *↑Methanobrevibacter*  *↑Akkermansia*  *↑Sutterella* | *- data not shown* | -microbiome alterations in MS include increases in *Methanobrevibacter* and *Akkermansia* and decreases in *Butyricimonas*, and correlate with variations in the expression of genes involved in dendritic cell maturation, interferon signalling and NF-kB signalling pathways in circulating T cells and monocytes  - increased abundances of *Prevotella* and *Sutterella*, decreased *Sarcina* in patients on disease-modifying treatment compared with untreated patients | [225] |
|  | Cohort: 70 individuals (20 MS patients, 50 healthy controls) |  |  | *↓Bacteroides*  *↓Faecalibacterium*  *↓Prevotella ↓Anaerostipes*  *↓Sutterella*  *↑Clostridium*  *↑Bifidobacterium ↑Streptococcus*  *↑Eggerthella* | *- data not shown* | - moderate dysbiosis in MS patients  - phylogenetic tree analysis - none of the significantly reduced clostridial species of MS patients overlapped with other spore-forming clostridial species able to induce colonic regulatory T cells, which prevent autoimmunity and allergies | [287] |
| **Epilepsy** | Cohort: 156 individuals (42 drug-resistant epilepsy patients, 49 drug-sensitive epilepsy patients, 65 healthy controls) | Abdominal pain, constipation, abdominal discomfort | Recurrent seizures,  neuro-inflammation, cell death, altered neurogenesis, sleep disturbances | *↑Bacteroides ↑Barnesiell*  *↑Clostridium ↑Atopobium ↑Holdemania*  *↑Dorea ↑Saccharibacteria ↑Delftia ↑Coprobacillus ↑Paraprevotella ↑Ruminococcus ↑Gemmiger ↑Akkermansia ↑Neisseria ↑Coprococcus ↑Fusobacterium ↑Methanobrevibacter ↑Phascolarctobacterium*  *↑Roseburia* | *- data not shown* | - gut microbial community of drug-resistant epilepsy was significantly altered with an abnormal increased abundance of rare flora  - gut microbiota composition of drug-sensitive epilepsy was similar with that of healthy controls  - patients with four seizures per year or fewer showed increased *Bifidobacteria* and *Lactobacillus* than those with more than four seizures per year | [229] |
|  | Cohort: 40 individuals (30 epilepsy patients, 10 healthy controls) |  |  | *↓Blautia*  *↓Coprococcus ↓Faecalibacterium ↓Ruminococcus*  *↓Bacteroides ↓Parabacteroides*  *↓Bifidobacterium ↓Collinsella*  *↑Campylobacter ↑Delftia*  *↑Haemophilus ↑Lautropia ↑Neisseria*  *↑Leptotrichia ↑Fusobacterium* | *- data not shown* | -Proteobacteria phylum - higher in patients with epilepsy  -Fusobacteria phylum - detected in 10.6% of the patients with epilepsy but not in the healthy group  - taxonomic drift and significant differences in intestinal microbiota of epilepsy-affected patients - autoimmune mechanisms and inflammation may have a role in the etiology of epilepsy | [235] |

**References**

[82] Vascellari S, Palmas V, Melis M, Pisanu S, Cusano R, Uva P, et al. Gut Microbiota and Metabolome Alterations Associated with Parkinson’s Disease. mSystems. 2020;5(5):e00561-20.

[93] Aizawa E, Tsuji H, Asahara T, Takahashi T, Teraishi T, Yoshida S, et al. Possible association of Bifidobacterium and Lactobacillus in the gut microbiota of patients with major depressive disorder. Journal of affective disorders. 2016;202:254-7.

[99] Valles-Colomer M, Falony G, Darzi Y, Tigchelaar EF, Wang J, Tito RY, et al. The neuroactive potential of the human gut microbiota in quality of life and depression. Nature Microbiology. 2019;4(4):623-32.

[105] Jiang H, Ling Z, Zhang Y, Mao H, Ma Z, Yin Y, et al. Altered fecal microbiota composition in patients with major depressive disorder. Brain, Behavior, and Immunity. 2015;48:186-94.

[107] Naseribafrouei A, Hestad K, Avershina E, Sekelja M, Linløkken A, Wilson R, et al. Correlation between the human fecal microbiota and depression. Neurogastroenterology & Motility. 2014;26(8):1155-62.

[116] Chen Y-h, Bai J, Wu D, Yu S-f, Qiang X-l, Bai H, et al. Association between fecal microbiota and generalized anxiety disorder: Severity and early treatment response. Journal of affective disorders. 2019;259:56-66.

[121] Jiang HY, Zhang X, Yu ZH, Zhang Z, Deng M, Zhao JH, et al. Altered gut microbiota profile in patients with generalized anxiety disorder. Journal of psychiatric research. 2018;104:130-6.

[125] Painold A, Mörkl S, Kashofer K, Halwachs B, Dalkner N, Bengesser S, et al. A step ahead: Exploring the gut microbiota in inpatients with bipolar disorder during a depressive episode. Bipolar disorders. 2019;21(1):40-9.

[131] Evans SJ, Bassis CM, Hein R, Assari S, Flowers SA, Kelly MB, et al. The gut microbiome composition associates with bipolar disorder and illness severity. Journal of psychiatric research. 2017;87:23-9.

[172] Zhu F, Ju Y, Wang W, Wang Q, Guo R, Ma Q, et al. Metagenome-wide association of gut microbiome features for schizophrenia. Nature Communications. 2020;11(1):1612.

[178] Shen Y, Xu J, Li Z, Huang Y, Yuan Y, Wang J, et al. Analysis of gut microbiota diversity and auxiliary diagnosis as a biomarker in patients with schizophrenia: A cross-sectional study. Schizophrenia research. 2018;197:470-7.

[191] Scheperjans F, Aho V, Pereira PAB, Koskinen K, Paulin L, Pekkonen E, et al. Gut microbiota are related to Parkinson's disease and clinical phenotype. Movement disorders. 2015;30(3):350-8.

[203] Vogt NM, Kerby RL, Dill-McFarland KA, Harding SJ, Merluzzi AP, Johnson SC, et al. Gut microbiome alterations in Alzheimer’s disease. Scientific Reports. 2017;7(1):13537.

[211] Stadlbauer V, Engertsberger L, Komarova I, Feldbacher N, Leber B, Pichler G, et al. Dysbiosis, gut barrier dysfunction and inflammation in dementia: a pilot study. BMC Geriatrics. 2020;20(1):248.

[214] Saji N, Murotani K, Hisada T, Kunihiro T, Tsuduki T, Sugimoto T, et al. Relationship between dementia and gut microbiome-associated metabolites: a cross-sectional study in Japan. Scientific Reports. 2020;10(1):8088.

[225] Jangi S, Gandhi R, Cox LM, Li N, von Glehn F, Yan R, et al. Alterations of the human gut microbiome in multiple sclerosis. Nature Communications. 2016;7(1):12015.

[229] Peng A, Qiu X, Lai W, Li W, Zhang L, Zhu X, et al. Altered composition of the gut microbiome in patients with drug-resistant epilepsy. Epilepsy research. 2018;147:102-7.

[235] Şafak B, Altunan B, Topçu B, Eren Topkaya A. The gut microbiome in epilepsy. Microbial Pathogenesis. 2020;139:103853.

[280] Coello K, Hansen TH, Sørensen N, Munkholm K, Kessing LV, Pedersen O, et al. Gut microbiota composition in patients with newly diagnosed bipolar disorder and their unaffected first-degree relatives. Brain, Behavior, and Immunity. 2019;75:112-8.

[281] Strati F, Cavalieri D, Albanese D, De Felice C, Donati C, Hayek J, et al. New evidences on the altered gut microbiota in autism spectrum disorders. Microbiome. 2017;5(1):24.

[282] Zhang M, Ma W, Zhang J, He Y, Wang J. Analysis of gut microbiota profiles and microbe-disease associations in children with autism spectrum disorders in China. Scientific Reports. 2018;8(1):13981.

[283] Tomova A, Husarova V, Lakatosova S, Bakos J, Vlkova B, Babinska K, et al. Gastrointestinal microbiota in children with autism in Slovakia. Physiology & Behavior. 2015;138:179-87.

[284] Li S, Zhuo M, Huang X, Huang Y, Zhou J, Xiong D, et al. Altered gut microbiota associated with symptom severity in schizophrenia. PeerJ. 2020;8.

[285] Lin A, Zheng W, He Y, Tang W, Wei X, He R, et al. Gut microbiota in patients with Parkinson's disease in southern China. Parkinsonism & Related Disorders. 2018;53:82-8.

[286] Nagpal R, Neth BJ, Wang S, Mishra SP, Craft S, Yadav H. Gut mycobiome and its interaction with diet, gut bacteria and Alzheimer's disease markers in subjects with mild cognitive impairment: A pilot study. EBioMedicine. 2020;59.

[287] Miyake S, Kim S, Suda W, Oshima K, Nakamura M, Matsuoka T, et al. Dysbiosis in the Gut Microbiota of Patients with Multiple Sclerosis, with a Striking Depletion of Species Belonging to Clostridia XIVa and IV Clusters. PLOS ONE. 2015;10(9):e0137429.
